# Investigating Intermolecular Interactions in a DME-Based Hybrid Ionic Liquid Electrolyte by HOESY NMR

**DOI:** 10.3389/fchem.2019.00004

**Published:** 2019-01-29

**Authors:** Derick Gyabeng, Pierre-Alexandre Martin, Urbi Pal, Michaël Deschamps, Maria Forsyth, Luke A. O'Dell

**Affiliations:** ^1^Institute for Frontier Materials, Deakin University, Geelong, VIC, Australia; ^2^CEMHTI, CNRS UPR 3079, Orléans University, Orléans, France

**Keywords:** ionic liquid electrolytes, ion interactions, Nuclear Overhauser Effect, HOESY NMR, cross-relaxation rates

## Abstract

The intermolecular interactions in a hybrid electrolyte based on various compositions of the ionic liquid *N*-methyl-*N*-propyl pyrrolidinium bis-fluorosulfonylimide (C_3_mpyrFSI), LiFSI salt and an ether-based additive, 1,2-dimethoxy ethane (DME), have been investigated using the HOESY (Heteronuclear Overhauser Effect SpectroscopY) NMR experiment. This NMR technique allows a quantification of the intermolecular interactions in ionic liquids (ILs) by measuring the cross-relaxation rate (σ) between different pairs of nuclei. Thereby, we compare the cross-relaxation rates between the cations, anions and DME in these hybrid electrolyte systems using ^1^H-^7^Li and ^1^H-^19^F HOESY experiments, and interpret the measured parameters in terms of ionic and molecular associations. The results give insights into the local coordination environment of the Li^+^ cations and their solvation by the FSI anions and DME.

## Introduction

Ionic liquids (ILs), typically consisting of an organic cation and an inorganic anion, have gained a lot of attention from researchers over the last two decades (Weingärtner, 2013). ILs are generally liquids below 100°C and have shown a lot of promise as electrolytes in batteries and other energy storage applications due to their versatile properties including low volatility, high thermal and electrochemical stability as well as high ionic conductivity (Macfarlane et al., 2014). Lithium-ion batteries play a very crucial role among current energy storage technologies due to their high energy and power densities (Bruce et al., 2012). They are commonly used in consumer electronics and increasingly in electric vehicles, and hence are very important in addressing global energy concerns. However, current lithium ion battery technologies still pose a safety risk primarily due to the volatile and flammable nature of the commonly used organic electrolytes (Wongittharom et al., 2014; Zhou et al., 2017). IL electrolytes are promising materials to replace these battery electrolytes in particular because of their low volatility and non-flammability. However, these properties typically coincide with lower ionic conductivities compared to traditional electrolyte systems.

In order to advance the design of ILs with improved ionic conductivities for battery applications, their molecular interactions need to be understood (Damodaran, 2016). The location and local environment of Li^+^ relative to the other ions in the electrolyte will play a very important role in the charge transport mechanisms and other processes occurring in the electrolyte (Martin et al., 2018b), which will in turn be major factors in determining the overall performance of the device. It is therefore vital to understand the molecular level structure and dynamics of ILs in order to improve their properties by tailoring their structure and composition.

Nuclear magnetic resonance (NMR) spectroscopy is a useful tool that has helped researchers to probe the structure and dynamics of ionic liquids. Pulsed field gradients (Pope et al., 2016; Hilder et al., 2017), relaxation measurements (Han et al., 2012; Filippov et al., 2015), and dynamic nuclear polarization (Sani et al., 2018) are some NMR techniques that have been used to investigate the structure and dynamics of ILs. Nuclear Overhauser Effect (NOE) based experiments are particularly useful to probe nanostructured arrangements in ILs through the transfer of nuclear spin polarization by cross-relaxation (Damodaran, 2016). In particular, the Heteronuclear Overhauser Effect SpectroscopY (HOESY) pulse sequence (Lingscheid et al., 2012; Bai et al., 2013; Chiappe et al., 2013; Castiglione et al., 2014, 2015; Giernoth et al., 2014; Tripathi and Saha, 2014) has been used to probe the various molecular interactions in ILs and their interactions with other solvents.

In our recent work on HOESY (Martin et al., 2018b), we demonstrated the importance of taking into account both the longitudinal relaxation times and self-diffusion coefficients of the nuclear spins when fitting HOESY build-up curves, as well as the normalization of the measured signals to the equilibrium magnetization. This analysis allows the quantitative comparison of NOEs (quantified as cross-relaxation rates) among different ILs, concentrations or temperatures. There have however been controversies regarding the interpretation of the intermolecular NOEs measured using this technique, owing to the fact that the internuclear distances are dependent on both the rotational and translational dynamics of the ions. Gabl et al. (2013) proposed that intermolecular NOEs measured between nuclear spins with similar frequencies, such as ^1^H and ^19^F, are sensitive to longer distances than nuclear spins whose frequencies are far apart. Our very recent work corroborates this prediction (Martin et al., 2018a), demonstrating the validity of interpreting intermolecular NOEs in terms of distances provided that such effects are taken into consideration.

Herein, we apply the HOESY NMR technique to a class of hybrid ionic liquid electrolytes recently reported (Pal et al., 2018) by our group that shows promise for applications in lithium electrochemistry with low polysulfide dissolution that is of importance for Li-S batteries. The addition of DME in these hybrid electrolytes has been shown to increase the relative mobility of the lithium ion through the formation of a chelate compound with lithium. Further understanding of the structure and dynamics in these electrolytes is of interest in terms of their future development. Herein, we quantitatively measure the Overhauser cross-relaxation rates in this DME-based hybrid ionic liquid electrolyte system for the intermolecular heteronuclear spin pairs ^1^H-^19^F and ^1^H-^7^Li. The results provide insights into the ionic associations and lithium cation solvation environment via the interactions between the Li^+^/FSI^−^ and the different hydrogen environments on the pyrrolidinium cations and DME molecules.

## Experimental Details

### Sample Preparation

**Figure 1:**
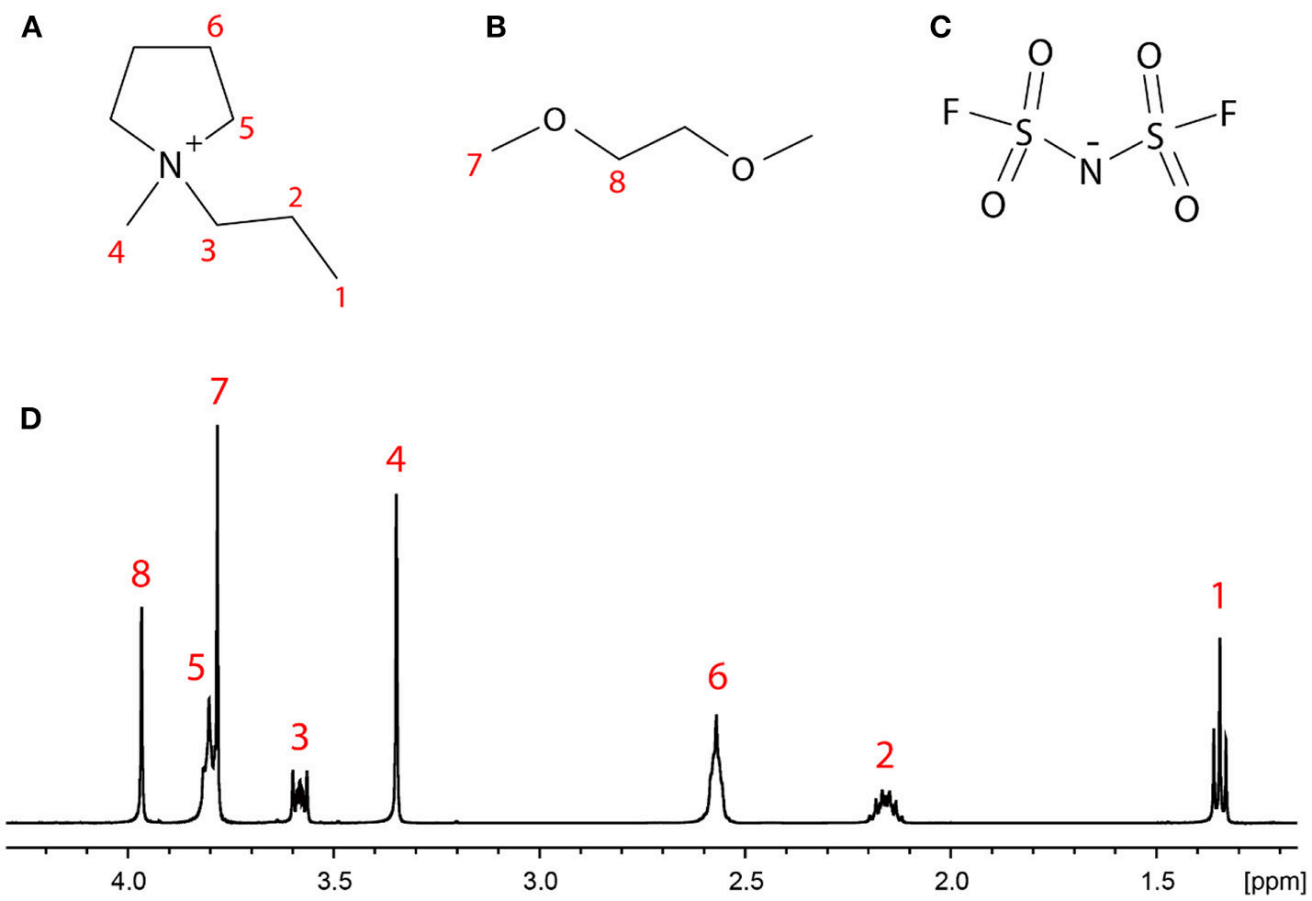
Structures of **(A)** the C_3_mpyr^+^ cation and **(B)** the DME molecule, with the eight groups of distinct protons labeled, **(C)** the FSI^−^ anion, and **(D)** the ^1^H NMR spectrum of LiDME-0.5 with peak assignment.

The ionic liquid *N*-methyl-*N*-propyl pyrrolidinium bis-fluorosulfonylimide (C_3_mpyrFSI) was purchased from Solvionic (France) with 99.5% purity, 1,2-dimethoxy ethane (DME) was purchased from Sigma-Aldrich with 99.9% purity (anhydrous), and the Li salt of bis-fluorosulfonylimide (LiFSI) was purchased from Nippon Shokubai (Japan) with 99.5% purity and used without further purification. The structures of these compounds are shown in Figure 1. These components were mixed together at different molar ratios as specified in Table 1. A neat sample of the C_3_mpyrFSI IL was also studied. All the materials were stored and packed in an Argon filled glove box. For the NMR experiments, the samples were packed in 3 mm capillary tubes inside the glove box to avoid contamination with water. The samples were then flame-sealed externally and inserted into 5 mm NMR tubes with D_2_O as outer layer for field lock and to minimize thermal convection effects.

**Table 1:** Relative molar ratios for the various ions in the hybrid electrolytes studied.

**Sample**	**Li^**+**^**	**FSI^**−**^**	**C_**3**_mpyr^**+**^**	**DME**	**Li:DME**
LiDME-0	0.25	0.50	0.25	0	1:0
LiDME-0.5	0.25	0.50	0.25	0.125	1:0.5
LiDME-1	0.25	0.50	0.25	0.25	1:1

### Diffusion Coefficient Measurements

Self-diffusion measurements for ^1^H, ^7^Li, and ^19^F were performed at 20°C using a pulsed field gradient stimulated echo NMR pulse sequence with a Bruker Avance III 7.05 T spectrometer equipped with a 5 mm Bruker Diff50 probe with a maximum gradient strength of 3,000 G/cm. The gradient pulse length used in the experiment was 2 ms and the diffusion time was 25 ms. To extract the diffusion coefficients, which are displayed in Table 2 below, the data were fitted in the Bruker Topspin software using the Stejskal-Tanner equation:

(1) $$\begin{array}{r} I=I_{o}\;\text{exp }\left(-\text{D}\gamma^{2}{\text{g}}^{2}\delta^{2}\left(\Delta -\frac{\delta }{3}\right)\right) \end{array}$$

where, *I* is the observed signal intensity, *I*_*o*_ is the maximum signal intensity, γ is the gyromagnetic ratio of the nucleus under observation, *g* is the gradient strength, δ is the gradient pulse duration and Δ is the diffusion time.

**Table 2:** Diffusion coefficient values of the various species in the pure IL and hybrid electrolytes, measured at 20°C.

	***D*** **(×10**^****−11****^**)/m**^****2****^**.s**^****−1****^			
**Sample**	**^**1**^H (C_**3**_mpyr^**+**^)**	**^**1**^H (DME)**	**^**7**^Li(Li^**+**^)**	**^**19**^F(FSI^**−**^)**
Neat C_3_mpyrFSI	2.63	–	–	3.11
LiDME-0	0.41	–	0.29	0.30
LiDME-0.5	0.96	0.85	0.83	0.93
LiDME-1	1.73	1.61	1.56	1.84

### Longitudinal Relaxation (*T_1_*) Measurements

Longitudinal relaxation times (*T*_1_) for ^1^H, ^7^Li, and ^19^F were measured at 20°C at a magnetic field strength of 11.7 T (500 MHz ^1^H frequency) using an inversion recovery pulse sequence and a Bruker Avance III spectrometer equipped with a 5 mm HX solution state probe. The data were fitted to an exponential curve using home-written Maple code.

### Cross-Relaxation Time Measurements

Cross-relaxation rates for ^7^Li-^1^H and ^19^F-^1^H for each sample were measured at 20°C using a ^1^H-detected HOESY pulse sequence, following an identical procedure to that previously reported (Martin et al., 2018a) and with the same NMR spectrometer and probe used for the longitudinal relaxation measurements. Mixing times for the HOESY build-up curves were varied over 22 values ranging from 3.7 ms to 4.5 s and 440 scans were recorded for each mixing time. A recycle delay between 5.0 and 6.0 s was used to ensure quantitative measurements. The HOESY build-up curves were fitted to the following expression with σ (the cross-relaxation rate) as the sole variable:

(2) $$\begin{array}{r} M\left(\tau \right)=M_{0}\sigma \frac{2\text{sinh}\left(\text{K}\tau \right)}{\text{K}}e^{\frac{-\left(R_{I}+R_{S}\right)\tau }{2}}e^{-\left({\text{D}}_{\text{I}}+{\text{D}}_{\text{S}}\right)\gamma_{\text{I}}\gamma_{\text{S}}{\text{g}}^{2}\delta^{2}\left(\Delta -\frac{\delta }{3}\right)} \end{array}$$

where M(τ) is the observed ^1^H signal, M_0_ is the thermal equilibrium magnetization of the ^7^Li or ^19^F spin, R_I/S_ are the longitudinal relaxation rates of the two spins, D_I/S_ are the diffusion coefficients of the ions containing the spins, γ_I/S_ are the nuclear gyromagnetic ratios, g is the gradient pulse strength, δ the gradient pulse length, Δ the delay time between the first and last gradient pulses, and:

(3) $$K=\frac{\sqrt{R_{I}^{2}-2R_{I}R_{S}+R_{S}^{2}+4\sigma^{\text{2}}}}{2}$$

A complete description of the signal normalization and HOESY curve fitting procedure can be found in our previous publication (Martin et al., 2018b).

## Results and Discussion

### ^7^Li-^1^H Cross-Relaxation Rates

The single-pulse excitation ^1^H NMR spectrum of the LiDME-0.5 sample is shown in Figure 1D with the peaks assigned according to the structures in Figures 1A,B. All of the peaks are well-resolved with the exception of sites 5 (the ring CH_2_ protons closest to the nitrogen on the C_3_mpyr cation) and 7 (the DME methyl protons), which show partial overlap. In the ^1^H-^7^Li HOESY experiments, the observed signals were much weaker, and the HOESY signal from site 5 was found to be generally less intense than that from site 7 in most cases. This made the extraction of the ^1^H-^7^Li cross-relaxation rates of the protons of site 5 from the DME-containing samples extremely difficult. The peak at around 3.8 ppm in the HOESY spectra was therefore fitted to a single cross-relaxation rate assumed to reflect only that of the site 7 protons on the DME molecule. For the ^1^H-^19^F HOESY experiments, higher signal intensities were obtained and these peaks were therefore able to be deconvoluted with separate HOESY build-up curves fitted for sites 5 and 7.

**Figure 2:**
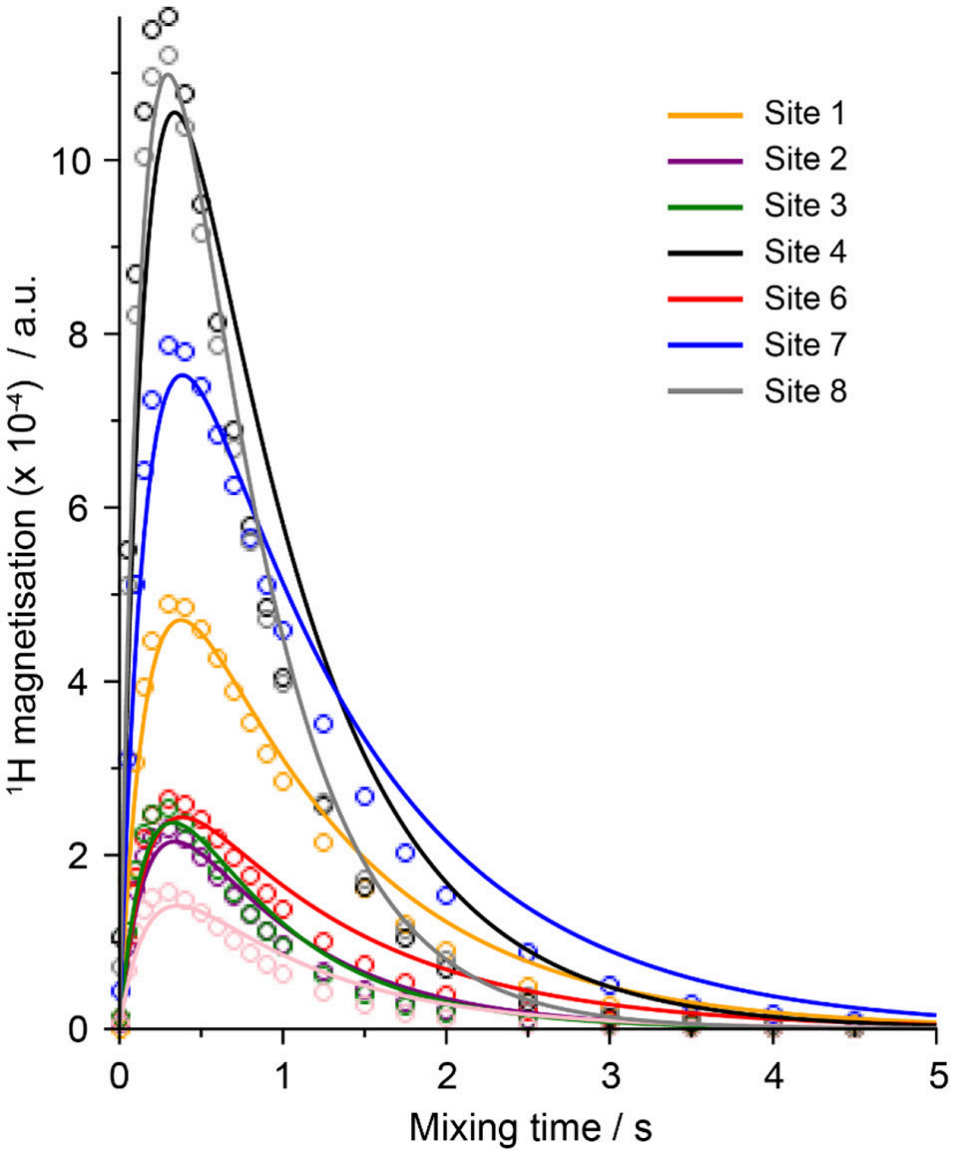
Fits of the ^1^H-^7^Li HOESY build-up curves from all of the ^1^H peaks of sample LiDME-0.5 at 20°C (site numbers refer to the structures/peaks shown in Figure 1).

The ^7^Li-^1^H cross-relaxation rates (×10^−4^ s^−1^) measured at 20°C from all three samples are presented in Table 3, and example fitted HOESY build-up curves from which these parameters were extracted are shown in Figure 2. The larger the value of the cross-relaxation rate, the stronger the NOE interaction between the two spins and this is typically interpreted as a closer average distance between the two nuclei. However, it should be noted here that variations in dynamics (specifically of the internuclear vector) will also play a role in determining the σ-value.

**Table 3:** ^7^Li-^1^H cross-relaxation rates measured from the hybrid electrolytes at 20°C.

		^****1****^**H-**^****7****^**Li** **σ/10**^****−4****^ **s**^****−1****^		
**Peak number**	**Site**	**LiDME-0**	**LiDME-0.5**	**LiDME-1**
1	Cation N-CH_2_-CH_2_-**CH**_**3**_	0.46	2.04	1.52
2	Cation N-CH_2_-**CH**_**2**_-CH_3_	0.44	0.41	0.35
3	Cation N-**CH**_**2**_-CH_2_-CH_3_	0.43	0.36	0.27
4	Cation N-**CH**_**3**_	1.79	1.82	1.41
5	Cation ring N-**CH**_**2**_-CH_2_	0.51	^*^	^*^
6	Cation ring N-CH_2_-**CH**_**2**_	0.56	0.66	0.51
7	DME **CH**_**3**_	–	5.41	18.5
8	DME **CH**_**2**_	–	16.0	37.2

*Uncertainties in these values are estimated at ± 10%*.

* *Values for peak 5 could not be obtained from the samples containing DME due to low intensity and overlap with peak 7*.

Considering first the interaction between the Li^+^ and the C_3_mpyr cation in sample LiMDE-0, the largest NOE is observed between the ^7^Li and the methyl proton on the carbon directly attached to the nitrogen site. This is consistent with a previous DNP NMR and molecular dynamics (MD) study of a glassy-state LiFSI-containing C_3_mpyrFSI sample in which measured C-Li distances suggested that the lithium cations are preferentially located on the side of the pyrrolidinium ring with this methyl group (Sani et al., 2018). The smallest NOEs are observed for the protons on the propyl chain. Interestingly, upon addition of DME to this system, the NOEs between the lithium and the various sites on the C_3_mpyr cation remain roughly the same, with the notable exception of the terminal methyl protons on the propyl chain (site 1), which show a significant increase in the cross relaxation rate.

With regards to the interactions between the Li^+^ cations and DME molecules, the σ-values in Table 3 show Li-DME NOEs that are larger by an order of magnitude or more than the Li-C_3_mpyr NOEs. This could be expected given the solvation of the Li^+^ cations by the ether oxygen groups of the DME molecules, as well as the fact that DME is a neutral molecule and hence will not show any charge repulsion from the Li. LiDME-1 shows the highest Li-DME NOEs and the lowest Li-C_3_mpyr NOEs (with the exception of the propyl CH_3_ group). It can be noted here that the diffusion coefficients of the Li^+^ and FSI^−^ ions are very similar in LiDME-0 (Table 2) indicating strong solvation of the lithium cations by the anions, but with the addition of DME the diffusion coefficient of the Li^+^ is closer to that of the DME molecules while the FSI anions diffuse faster. In combination with the HOESY results, this suggests that the DME solvates the Li^+^ by displacing (at least partially) the FSI^−^ ions. The solvation of the Li^+^ by the DME molecules and the displacement of the FSI anions around the Li^+^ means that there will be more repulsion between the Li and the C_3_mpyr (e.g., a Li-DME-FSI cluster will have a net neutral charge compared to an ${\text{Li}(\text{FSI})}_{2}^{-}$ cluster that will be more attracted to the C_3_mpyr), and this can explain the reduced Li-C_3_mpyr NOEs as the DME concentration increases. It was not possible to obtain reliable ^7^Li-^1^H cross-relaxation rates for the CH_2_ on the IL cation ring near N^+^ because it strongly overlaps with the terminal DME proton peak. Finally, the Li-DME NOEs are stronger for the CH_2_ protons on the DME molecule than for the CH_3_ protons. This may reflect the conformation of the DME molecule when solvating the lithium, however, the different dynamics of the two groups of protons (e.g., the rotation of the methyl group) may also play a role in the different NOE efficiencies.

### ^19^F-^1^H Cross-Relaxation Rates

The cross-relaxation rates extracted from ^19^F-^1^H HOESY build-up curves are displayed in Table 4, including values obtained from the neat C_3_mpyrFSI IL (without LiFSI) for comparison. These values give insights into the interactions between the FSI anions and the C_3_mpyr^+^ cation/DME species, and once again we make the assumption that larger NOEs reflect closer average distances between these anions and the different proton sites. In the neat IL, the ^19^F-^1^H cross-relaxation rate is largest for the N-CH_3_ group and the values decrease as the proton sites become more distant from this central nitrogen site. This can be interpreted in terms of the localized positive charge at this region of the cation which acts to attract the FSI anions. Upon addition of the LiFSI salt (i.e., sample LiDME-0), the σ-values increase for all proton sites. Rather than a net increase in the FSI-C_3_mpyr interaction strength, we interpret this as being due to a change in the overall dynamics of the system, as it is well known that adding lithium salts to ionic liquids results in a significant increase in viscosity (Bose et al., 2018). This phenomenon is confirmed by the decrease in diffusion coefficients of both ions by around an order of magnitude upon the addition of LiFSI salt in LiDME-0 as shown in Table 2. The LiDME-0 sample also shows the largest ^19^F-^1^H NOE for the N-CH_3_ protons which is again consistent with the picture established by DNP NMR and MD simulations (Sani et al., 2018) of a negatively charged Li(FSI)_n_ cluster existing close to this part of the cation.

**Table 4:** ^1^H-^19^F cross-relaxation rates measured from the neat C_3_mpyrFSI ionic liquid and the three hybrid electrolyte samples at 20°C.

**Peak number**	**Site**	^****1****^**H-**^****19****^**F** **σ/10**^****−4****^ **s**^****−1****^			
		**Neat IL**	**LiDME-0**	**LiDME-0.5**	**LiDME-1**
1	Cation N-CH_2_-CH_2_-**CH**_**3**_	1.67	8.62	7.79	2.46
2	Cation N-CH_2_-**CH**_**2**_-CH_3_	3.05	7.64	4.34	1.91
3	Cation N-**CH**_**2**_-CH_2_-CH_3_	3.81	8.04	5.14	2.41
4	Cation N-**CH**_**3**_	16.1	16.7	17.5	7.92
5	Cation ring N-**CH**_**2**_-CH_2_	3.51	6.48	2.67	1.27
6	Cation ring N-CH_2_-**CH**_**2**_	1.12	6.39	3.97	1.27
7	DME **CH**_**3**_	–	–	21.8	10.09
8	DME **CH**_**2**_	–	–	38.4	12.02

*Uncertainties in these values are estimated at ± 10%*.

Upon addition of DME to this system, the σ-values can be seen to decrease overall, and once again this can be attributed at least in part to a reduction in the sample viscosity and an increase in the overall dynamics of the liquid (reflected also by an increase in the diffusion coefficients in Table 2). With regards to the FSI-C_3_mpyr interactions, the overall trends remain similar to the LiDME-0 sample with the strongest interaction still being between the FSI and the N-CH_3_ group. The fact that the interaction between the FSI anions and the terminal methyl group on the propyl chain of the cation does not appear to increase upon DME addition suggests that the increased interactions between the lithium cations and this particular group may be due to the presence of Li(DME)_n_ clusters. However, the much larger ^19^F-^1^H NOE values observed for the protons on the DME molecules strongly indicate the presence of lithium cations that are simultaneously solvated by both FSI and DME. MD simulations are currently being carried out on this system and should help with the further interpretation of these NOE values.

## Conclusions

We have measured ^7^Li-^1^H and ^19^F-^1^H Overhauser cross-relaxation rates from several pyrrolidinium-based ionic liquid electrolytes containing lithium salt and the 1,2-dimethoxy ethane additive in different ratios. These parameters allow a quantitative comparison of the interactions of the Li^+^ and FSI^−^ ions with the IL cation and DME molecules in the different electrolytes. Both FSI-C_3_mpyr and Li-C_3_mpyr NOEs decrease overall as the DME content increases. The Li-DME and FSI-DME NOEs were found to be the largest, indicating the solvation of Li^+^ by both of these species. Strong FSI-DME NOEs indicate simultaneous solvation of a Li^+^ by both DME and FSI. Li-C_3_mpyr and FSI-C_3_mpyr interactions were strongest for the N-CH_3_ protons which is consistent with a previous study that concluded that ${\text{Li}(\text{FSI})}_{\text{n}}^{-}$ clusters preferentially locate close to that region of the cation (Sani et al., 2018). The measured diffusion coefficients assisted greatly in interpreting the NOE values, providing additional evidence for ion associations while also reflecting composition-dependent changes in the overall dynamics that affect the sizes of the measured NOE values. These results help to quantify the ionic interactions and the role that the additive 1,2-dimethoxy ethane plays in the ionic liquid *N*-methyl-*N*-propyl pyrrolidinium bis-fluorosulfonylimide. Further measurements as a function of temperature, along with the correlation to molecular dynamics simulation data, will allow a more detailed interpretation of these cross-relaxation rates, and such work is currently underway.

## Author Contributions

DG carried out the experiments, data analysis and interpretation, and wrote the paper. P-AM, UP, MD, MF, and LO contributed to the interpretation of the results and edited the paper.

### Conflict of Interest Statement

The authors declare that the research was conducted in the absence of any commercial or financial relationships that could be construed as a potential conflict of interest.

## References

[B1] Bai T. Ge R. Gao Y. Chai J. Slattery J. M. (2013). The effect of water on the microstructure and properties of benzene/[Bmim][AOT]/[Bmim][BF4] microemulsions. Phys. Chem. Chem. Phys. 15, 19301–19311. 10.1039/c3cp53441c 24121764

[B2] Bose P. Deb D. Bhattacharya S. (2018). Ionic liquid based nanofluid electrolytes with higher lithium salt concentration for high-efficiency, safer, lithium metal batteries. J. Power Sour. 406, 176–184. 10.1016/j.jpowsour.2018.10.050

[B3] Bruce P. G. Freunberger S. A. Hardwick L. J. Tarascon J. M. (2012). Li–O_2_ and Li–S batteries with high energy storage. Nat. Mater. 11, 19–29. 10.1038/nmat3191 22169914

[B4] Castiglione F. Appetecchi G. B. Passerini S. Panzeri W. Indelicato S. Mele A. (2015). Multiple points of view of heteronuclear NOE: long range vs short range contacts in pyrrolidinium based ionic liquids in the presence of li salts. J. Mol. Liquids 210, 215–222. 10.1016/j.molliq.2015.05.036

[B5] Castiglione F. Famulari A. Raos G. Meille S. V. Mele A. Appetecchi G. B.. (2014). Pyrrolidinium-based ionic liquids doped with lithium salts: how does Li+ coordination affect its diffusivity? J. Phys. Chem. B 118, 13679–13688. 10.1021/jp509387r 25368963

[B6] Chiappe C. Sanzone A. Mendola D. Castiglione F. Famulari A. Raos G.. (2013). Pyrazolium- versus imidazolium-based ionic liquids: structure, dynamics and physicochemical properties. J. Phys. Chem. B 117, 668–676. 10.1021/jp3107793 23252760

[B7] Damodaran K. (2016). Recent NMR Studies of Ionic Liquids. Annual Reports on NMR Spectroscopy, 1st Edn., Vol. 88. London: Elsevier Ltd 10.1016/bs.arnmr.2015.11.002

[B8] Filippov A. Azancheev N. Taher M. Shah F. U. Rabét P. Glavatskih S. (2015). Self-diffusion and interactions in mixtures of imidazolium bis(mandelato)borate ionic liquids with polyethylene glycol: 1H NMR study. Magn. Reson. Chem. 53, 493–497. 10.1002/mrc.4232 25854162

[B9] Gabl S. Steinhauser O. Weingärtner H. (2013). From short-range to long-range intermolecular NOEs in ionic liquids: frequency does matter. Angew. Chem. Int. Edn. 52, 9242–9246. 10.1002/anie.201302712 23776138

[B10] Giernoth R. Bröhl A. Brehm M. Lingscheid Y. (2014). Interactions in ionic liquids probed by *in situ* NMR spectroscopy. J. Mol. Liquids 192, 55–58. 10.1016/j.molliq.2013.07.010

[B11] Han K. S. Li S. Hagaman E. W. Baker G. A. Cummings P. Sheng D. (2012). Rotational and translational dynamics of N -Butyl- N -methylpiperidinium trifluoromethanesulfonimide ionic liquids studied by NMR and MD simulations. J. Phys. Chem. C 116, 20779–20786. 10.1021/jp3069283

[B12] Hilder M. Gras M. Pope C. R. Kar M. Macfarlane D. R. Forsyth M.. (2017). Effect of mixed anions on the physicochemical properties of a sodium containing alkoxyammonium ionic liquid electrolyte. Phys. Chem. Chem. Phys. 19, 17461–17468. 10.1039/c7cp03318d 28650511

[B13] Lingscheid Y. Arenz S. Giernoth R. (2012). Heteronuclear NOE spectroscopy of ionic liquids Chem. Phys. Chem. 13, 261–266. 10.1002/cphc.201100622 22002917

[B14] Macfarlane D. R. Tachikawa N. Forsyth M. Pringle J. M. Howlett P. C. Elliott G. D. (2014). Energy applications of ionic liquids. Energy Environ. Sci. 7, 232–250. 10.1039/c3ee42099j

[B15] Martin P. A. Chen F. Forsyth M. Deschamps M. O'Dell L. A. (2018a). Correlating intermolecular cross-relaxation rates with distances and coordination numbers in ionic liquids. J. Phys. Chem. Lett. 9, 7072–7078. 10.1021/acs.jpclett.8b03021 30395468

[B16] Martin P. A. Salager E. Forsyth M. O'Dell L. A. Deschamps M. (2018b). On the measurement of intermolecular heteronuclear cross relaxation rates in ionic liquids. Phys. Chem. Chem. Phys. 20, 13357–13364. 10.1039/c8cp00911b 29718051

[B17] Pal U. Girard G. M. A. O'Dell L. A. Roy B. Wang X. Armand M. (2018). Improved Li-ion transport by DME chelation in a novel ionic liquid-based hybrid electrolyte for Li–S battery application. J. Phys. Chem. C 122, 14373–14382. 10.1021/acs.jpcc.8b03909

[B18] Pope C. R. Kar M. MacFarlane D. R. Armand M. Forsyth M. O'Dell L. A. (2016). Ion dynamics in a mixed-cation alkoxy-ammonium ionic liquid electrolyte for sodium device applications. Chem. Phys. Chem. 17, 3187–3195. 10.1002/cphc.201600692 27490422

[B19] Sani M. A. Martin P. A. Yunis R. Chen F. Forsyth M. Deschamps M.. (2018). Probing ionic liquid electrolyte structure via the glassy state by dynamic nuclear polarization NMR spectroscopy. J. Phys. Chem. Lett. 1007–1011. 10.1021/acs.jpclett.8b00022 29420892

[B20] Tripathi N. Saha S. (2014). Unraveling the heterogeneity in N Butyl-N-methylpiperidinium trifluromethanesulfonimide ionic liquid by 1D and 2D NMR spectroscopy. Chem. Phys. Lett. 607, 57–63. 10.1016/j.cplett.2014.05.046

[B21] Weingärtner H. (2013). NMR studies of ionic liquids: structure and dynamics. Curr. Opin. Coll. Interface Sci. 18, 183–189. 10.1016/j.cocis.2013.04.001

[B22] Wongittharom N. Lee T. C. Wang C. H. Wang Y. C. Chang J. K. (2014). Electrochemical performance of Na/NaFePO4 sodium-ion batteries with ionic liquid electrolytes. J. Mater. Chem. A 2:5655 10.1039/c3ta15273a

[B23] Zhou Y. Wang X. Zhu H. Armand M. Forsyth M. Greene G. W.. (2017). N-ethyl-N-methylpyrrolidinium bis(fluorosulfonyl)imide-electrospun polyvinylidene fluoride composite electrolytes: characterization and lithium cell studies. Phys. Chem. Chem. Phys. 19, 2225–2234. 10.1039/c6cp07415d 28054060

